# Approach of Family Physicians to Pediatric Eye Screening in Diyarbakır

**DOI:** 10.4274/tjo.galenos.2018.10829

**Published:** 2019-02-28

**Authors:** Zeynep Gürsel Özkurt, Selahattin Balsak, Mehmet Sinan Çamçi, Kadir Bilgen, İbrahim Halil Katran, Adar Aslan, Çağla Çilem Han

**Affiliations:** 1 Dicle University Faculty of Medicine, Department of Ophthalmology, Diyarbakır, Turkey; 2 University of Health Sciences, Gazi Yaşargil Training and Research Hospital, Ophthalmology Clinic, Diyarbakır, Turkey

**Keywords:** Red reflex test, eye screening, congenital cataract, retinoblastoma, negative performance

## Abstract

**Objectives:**

In Turkey, preventive medicine services are the responsibility of family physicians and vision screening is a key component of this responsibility. In this study, we aimed to investigate the approach of family physicians to vision screening in infants and children.

**Materials and Methods:**

Data were collected using a 16-item questionnaire administered to 100 family physicians working in the center and provinces of Diyarbakır.

**Results:**

The results indicated that 88 (88%) physicians declared knowing what the red reflex test was, while 12 physicians declared that they had never heard of it. Only 16 (16%) physicians performed the test routinely and 36 (36%) physicians performed it only in suspicious cases. Ten (10%) physicians indicated that they did not refer the patients to an ophthalmologist even though they did not perform the red reflex test. Moreover, 5 (5%) physicians did not have an ophthalmoscope and 12 (12%) physicians reported not knowing how to use an ophthalmoscope. Forty (40%) of the physicians measured preschool visual acuity at least once. Sixty-six (66%) physicians referred younger children who could not express their vision problems to an ophthalmologist. Four (4%) physicians declared that they would delay surgery in children with strabismus until they were old enough for surgery. Ninety-three (93%) physicians suggested that educational seminars about vision screening would be beneficial.

**Conclusion:**

Educational seminars about vision screening may have favorable outcomes. The medical devices in family medicine centers should be improved. Vision screening can be added to the negative performance-based compensation system in order to increase physicians’ attention to vision screening. To implement detailed eye screening programs like those in developed countries, an infrastructure should be established for this screening program.

## Introduction

The practice of family medicine in Turkey was first initiated in Düzce in 2005, and its nationwide expansion began as of 2010. The duties and responsibilities of family physicians are defined as approaching registered individuals holistically and providing individualized preventive, therapeutic, and rehabilitative health services as part of a team. Preventive medical services personally provided by family physicians include vaccination, pregnancy follow-up, and infant/child care.^1^

Examination of the eye and adnexa is an important component of infant/child care. Red reflex examination is a simple, non-invasive screening test that can easily be performed by family physicians. The red reflex occurs when light from the ophthalmoscope passes through the transparent structures of the eye and is reflected by the fundus back to the eye of the examiner.^2^ This test enables early diagnosis of important and treatable sight- and life-threatening diseases such as congenital cataracts and retinoblastoma.^3^ In their statement issued in 2016, the American Academy of Pediatrics recommended performing red reflex examination at every physician visit for babies aged 0-6 months, and again at 6 months, 12 months, 1-3 years, 4-5 years, and 6 years old.^4^

In childhood, eye problems such as refractive errors, amblyopia, and strabismus may result in functional and preventable visual impairment or even blindness. Vision screening conducted at schools in Southeastern Anatolia in 2013 revealed that 10.6% of the children had refractive errors that required correction and were unrecognized by the children themselves. In the same study, amblyopia was detected in 2.6% of children, and the most frequent causes of ambylopia were reported as anisometropia and strabismus.^5^ These preventable causes of visual impairment are issues that should be addressed by the family physicians in pediatric ophthalmology follow-up visits as part of preventive medicine.

The Southeastern Anatolia region includes the lowest ranked cities in Turkey in terms of socio-economic development.^6^ It is especially important for family physicians to exercise due diligence in the practice of preventive medicine in this region. In this study, we conducted a survey of family physicians working in the province of Diyarbakır. Our aim was to analyze the approach of physicians in this region to eye screening tests for infants and children, evaluate how knowledgeable and equipped they are, and to determine whether they require continuing education on this topic.

## Materials and Methods

The study protocol was approved by Diyarbakır Gazi Yaşargil Training and Research Hospital Ethics Committee, and the study was carried out in accordance with the principles of the Declaration of Helsinki. A questionnaire consisting of 16 items pertaining to eye screening tests for infants and children was created (Table 1). Questions were prepared based on internationally recommended pediatric eye screening tests. The aim was to determine to what degree these screening tests are known and performed by family physicians, evaluate their level of knowledge and awareness of the subject and the availability of necessary equipment, and learn their views on the need for further education on this topic. Of 492 family physicians working in the urban center and surrounding districts of the Diyarbakır province, 100 were contacted. The questionnaire was administered to the family physicians participating in the study. Data were recorded and the results were calculated as percentages.

## Results

One hundred family physicians working in the central and surrounding districts of the Diyarbakır province participated in this survey. When asked what the red reflex test is, 88 (88%) of the physicians said they knew of this test, while 12 (12%) stated they had never heard of it. When the 88 physicians who knew about the red reflex test were asked whether they perform it, 52 (52%) stated that they did red reflex examination, while 36 (36%) stated they had never performed it despite knowing of it. Only 16 (16%) of 52 physicians who performed the red reflex test stated that they did so regularly, while 36 (36%) reported that they performed it only in suspicious cases. Of these 52 physicians, 1 reported detecting an absent red reflex only once, and another reported detecting absent red reflex 3 times. Among all the physicians participating in the survey, the proportion who regularly performed red reflex examination was found to be 16%. Of the 36 physicians who never did red reflex screening, 10 (10%) stated that they did not refer infants to an ophthalmologist despite not performing the test. Thirty-three (33%) of the participating physicians were aware that the red reflex test should be performed in every infant examination. Seventy-two physicians (72%) stated that it would be beneficial to add the red reflex test to follow-up charts, just like height and weight measurements

The physicians were asked before what age (in months) congenital cataracts should be treated to avoid the development of amblyopia. Only 31 physicians (31%) responded correctly, while 69 physicians (69%) did not know about the timing of treatment even though they detect congenital cataracts. When asked whether their practices were equipped with a direct ophthalmoscope, 95 physicians (95%) indicated that they had an ophthalmoscope, while 5 physicians (5%) did not. Thirty-five (35%) of the physicians reported never using a direct ophthalmoscope, 12 (12%) of whom stated that they did not know how to use an ophthalmoscope. 

The physicians were asked questions about eye screening tests for pediatric patients. Forty (40%) of the physicians reported measuring children’s visual acuity in both eyes separately using a preschool chart. Sixty-six (66%) of the physicians referred children who were too young (1-4 years old) to describe their visual acuity using a chart to an ophthalmologist for refraction examination. When asked about their approach to strabismus in infants or children, 96 physicians (96%) stated that they would refer the patient directly to an ophthalmologist, and 4 physicians (4%) said they would take no action until the patient was old enough to undergo surgery.

When asked whether an educational seminar should be held for family physicians regarding eye screening tests for infants and children, 93 (93%) of the physicians answered positively and stated that they would like to attend if such a seminar was held.

Approximately half of the physicians did not want to share for how long they had been working as family physicians and whether they were general practitioners or specialists. As a result, the survey responses could not be compared based on these data.

## Discussion

Red reflex examination was first described by Bruckner in 1962 and was recognized as a method to screen for vision- and life-threatening eye diseases in children.^3^ The ophthalmoscope works on the principle that light entering the patient’s pupil passes through the transparent ocular structures and is reflected from the fundus back to the observer. When the cornea, aqueous humor, crystalline lens, and vitreous are transparent, the reflection will be red, yellow, orange, or a combination of these colors.^2^ In conditions that obstruct the passage of light (e.g., cataract) or prevent the proper reflection from the ocular fundus (e.g., retinoblastoma), the reflection will appear black, white, or nonhomogeneous.^3,7^

In 2016, the American Academy of Pediatrics recommended performing red reflex examination at every physician visit for babies aged 0-6 months, and again at 6 months, 12 months, between 1-3 years, 4-5 years, and at 6 years old.^4^ Red reflex screening protocols are followed in many developed countries. For instance, red reflex screening is performed in more than 90% of pediatric and neonatal units in Sweden and was reported to have tripled the detection rate of ocular pathologies (from 19% to 64%).^8^

In order to prevent visual impairment caused by congenital cataracts in newborns, surgery must be done within 6 weeks of birth.^9^ Detecting congenital cataracts this early is only possible if pediatricians or family physicians perform red reflex examination. In a study conducted in the United Kingdom, it was found that less than half of congenital cataracts detected between 1995-1996 were detected by screening before the age of 8 weeks.^10^ A study in the United States of America reported that 38% of congenital cataracts were detected after 6 weeks.^11^ Another disease that can be detected by red reflex examination is retinoblastoma. Retinoblastoma, the most common primary intraocular tumor in childhood, leads to leukocoria. A study on retinoblastoma determined that only 123 (8%) of 1831 children with leukocoria were detected by a pediatrician.^12^

There are no data in Turkey regarding what proportion of congenital cataract and retinoblastoma are detected by family physicians using red reflex examination. In the present study, family physicians were questioned about their knowledge and practice of red reflex screening. Twelve percent of the respondents stated that they had never heard of the red reflex test, while 36% stated that they knew of but had never performed it. Only 16% of the physicians performed red reflex screening regularly. Thirty-three (33%) physicians expressed that they knew this screening should be performed in every infant examination. Twelve physicians (12%) did not know how to use a direct ophthalmoscope. Even if able to detect congenital cataract, only 31% of the respondents were aware that the baby must undergo cataract surgery before the age of 6 weeks. Awareness of this issue must be raised among family physicians in Turkey to facilitate the timely detection of vision- and life-threatening diseases such as congenital cataracts and retinoblastoma. 

Eye screening during childhood is important to prevent amblyopia secondary to refractive error and strabismus. Legislative decree no: 633 issued in 2011 stated that the family physician is responsible for the overall health of school-age children and must provide diagnostic and therapeutic services for the health problems of school-age children.^13^ However, an eye screening program carried out in schools in Southeastern Anatolia in 2013, refractive errors that required glasses and were unnoticed by the child were detected in 10.6% of children, and amblyopia was detected in 2.6% of children. The two most frequent causes of amblyopia were found to be anisometropia and strabismus.^5^ In a study performed in Turkey in 2017, approximately 823 primary school children living in İstanbul underwent eye screening and it was reported that 22% of children in private schools and 65% of children in public schools had never had a vision test before. The authors also emphasized that previously unrecognized vision problems were twice as common in children from a lower socio-economic background.^14^ These two studies suggest that, despite the decree, the diagnostic and therapeutic services provided to school-age children by family physicians are inadequate in terms of eye health. In our study, 40 physicians (40%) measured the visual acuity of preschool children in both eyes separately using an appropriate chart. For the younger age groups, 66% of the physicians referred children to an ophthalmologist for refraction test. These results show that almost half of the children in the Diyarbakır province begin school without ever undergoing a refraction test. 

Strabismus can develop in the absence of an underlying cause or due to severe pathologies like retinoblastoma.^15^ When we asked the family physicians about their approach to children in whom they detected strabismus, 4 physicians (4%) stated that they would wait until the patient was old enough to undergo surgery. However, there is no specified age for strabismus surgery; therefore, making the patient wait can result in delayed treatment of potentially vision- and life-threatening pathologies. It is important to make physicians aware of this. 

Infant and child eye examinations are included in routine screening tests in developed countries. For example, visual acuity assessment has been a part of the screening program in the Netherlands since 1960. Every 5 years, the nurses and physicians responsible for the screening participate in a one-day training program led by a screening director and orthoptist. Patients have a total of 7 free eye screenings (at 1, 2, 3, 6-9, 14-24, 36, and 45 months of age). Examinations done between 1 and 4 months of age include inspection of the eye and adnexa, red reflex examination, Hirschberg test, and pupillary light reflex test. Examinations done between 6 and 24 months of age include inspection of the eye and adnexa, Hirschberg test, pupillary light reflex test, cover-uncover test, alternate cover test, and assessment of eye movement and monocular tracking. Visual acuity is measured using the Amsterdam Picture Chart at 36 months and the Landolt C graph at 45 months of age.^16^ While such detailed eye screening tests are standard in developed countries, there is still a conspicuous lack of regular screening programs in our country. 

In article 24 of the regulations governing the practice of family medicine issued in an Official Gazette in 2013, an ophthalmoscope and Snellen chart are included in the minimum necessary medical device and equipment list for family health centers.^17^ Of the physicians who participated in our survey, 5 (5%) stated that they did not have ophthalmoscope in their center. This indicates that family health centers are not being adequately inspected in terms of medical devices and equipment.

The concept of negative performance-based compensation in family medicine is defined in the Family Medicine Law no: 5258 issued in 2004, article 3, section 7 entitled “Personnel status and financial rights” as follows: In the event of incomplete provision of preventive medical services according to the standards determined by the Ministry of Health, up to 20% of the gross charge shall be deducted from payment”.^1^ Clause d of article 4 of this law specifies vaccination, pregnancy follow-up, and infant-child follow-up as preventive medical services that must be provided. Deficiencies in preventive medicine practices apart from these services are not included in the implementation of negative performance-based pay. We believe that pediatric eye screening must be included in the scope of negative performance in order to improve adherence to these guidelines.

## Conclusion

In conclusion, family physicians in Turkey must be better informed about which eye tests should be done for infants and children and at what ages they must be performed. Educational seminars can be held on this topic. Including infant and child eye screening tests in the negative performance-based compensation scheme will increase awareness of this subject. It is also important to begin establishing the infrastructure necessary to perform detailed ophthalmologic screening programs in our country.

## Figures and Tables

**Table 1:**
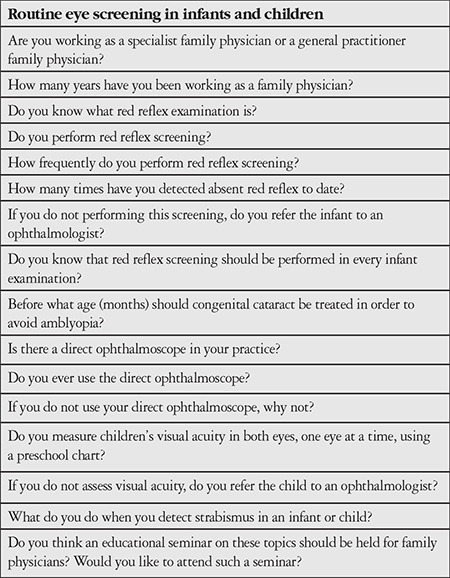
Survey questions
